# Inherent bacterial DNA contamination of extraction and sequencing reagents may affect interpretation of microbiota in low bacterial biomass samples

**DOI:** 10.1186/s13099-016-0103-7

**Published:** 2016-05-26

**Authors:** Angela Glassing, Scot E. Dowd, Susan Galandiuk, Brian Davis, Rodrick J. Chiodini

**Affiliations:** St. Vincent Healthcare, Sisters of Charity of Leavenworth Health System, Billings, MT USA; Department of Biological and Physical Sciences, Montana State University-Billings, 1500 University Drive, Billings, MT 59101 USA; Molecular Research Laboratory (Mr. DNA), Shallowater, TX USA; Hiram C. Polk, Jr. MD Department of Surgery, University of Louisville, Louisville, KY USA; Department of Surgery, Paul L. Foster School of Medicine, Texas Tech University Health Sciences Center, El Paso, TX USA

**Keywords:** Contamination, PCR, Metagenomics, Microbiota, Inflammatory bowel disease, 16S sequencing

## Abstract

**Background:**

The advent and use of highly sensitive molecular biology techniques to explore the microbiota and microbiome in environmental and tissue samples have detected the presence of contaminating microbial DNA within reagents. These microbial DNA contaminants may distort taxonomic distributions and relative frequencies in microbial datasets, as well as contribute to erroneous interpretations and identifications.

**Results:**

We herein report on the occurrence of bacterial DNA contamination within commonly used DNA extraction kits and PCR reagents and the effect of these contaminates on data interpretation. When compared to previous reports, we identified an additional 88 bacterial genera as potential contaminants of molecular biology grade reagents, bringing the total number of known contaminating microbes to 181 genera. Many of the contaminants detected are considered normal inhabitants of the human gastrointestinal tract and the environment and are often indistinguishable from those genuinely present in the sample.

**Conclusions:**

Laboratories working on bacterial populations need to define contaminants present in all extraction kits and reagents used in the processing of DNA. Any unusual and/or unexpected findings need to be viewed as possible contamination as opposed to unique findings.

**Electronic supplementary material:**

The online version of this article (doi:10.1186/s13099-016-0103-7) contains supplementary material, which is available to authorized users.

## Background

Microbes are the predominant life form on earth and probably have been the since prokaryotic life began on earth some 3.5 billion years ago [1, 2]. Since DNA may persist for thousands of years, it is not surprising that evidence of bacterial existence (bacterial DNA) can be found in almost all ecosystems. This has become more evident with the advent of highly sensitive molecular techniques, such as PCR, 16S rRNA gene and metagenomic shotgun sequencing methods, capable of identifying hosts of cultivable and uncultivable microorganisms. By the use of these methods, it has long been realized that molecular biology grade (MBG) reagents, DNA isolation kits, PCR master mixes, and several other laboratory supplies used in processing and analyses of DNA are contaminated with bacterial DNA [3–7]. Although microbial contamination from exogenous and endogenous sources is a constant worry of microbiologists, it is often less of a concern for molecular biologists who erroneously assume that MBG reagents are free of microbial DNA. As such, the potential impact of microbial contamination by MBG products that can distort taxonomic distributions and the relative frequencies observed in sequencing datasets is frequently overlooked.

We recently developed and reported on methods to effectively separate mucosal and submucosal intestinal tissues and compare the microbial populations of the mucosa to that of the subjacent submucosa [8]. Since bacterial translocation across the mucosal barrier is a prominent feature in Crohn’s disease [9], we sought to enumerate the bacterial load within submucosal tissues of resected tissues from patients with Crohn’s disease. However, the amount of contamination in negative no-template controls and in standards made quantitative determinations unachievable in submucosal samples, presumably a combination of low bacterial biomass and large amounts of competitive human DNA in the samples.

Although largely unappreciated, the scientific literature documents widespread microbial DNA contamination of PCR reagents and several methods had been proposed to eliminate and/or reduce background impurities and noise including UV irradiation, restriction endonuclease and DNAse digestion, and treatment with ethidium monoazide (EMA) [7, 10, 11], but none of the proposed methods has proved capable of reliably reducing DNA reagent contamination.

Despite reports and alleged common knowledge, reagent contamination apparently remains underappreciated in the microbiota research community. Most DNA sequence-based publications describing the microbial communities of low-biomass environments do not carry out sequencing of negative controls, or do not describe their contaminant removal or identification procedures [12]. A number of microbiota studies report taxa, often statistically noteworthy, that overlap with those reported for negative control reagents and water [12–14].

Previous reports dealing with issues of contamination used hypothetical situations and spiked samples to determine the effects of contamination. Herein we report on our efforts to determine bacterial loads within submucosal intestinal tissues and blood and the effects of contamination on those efforts. We also report the identification of bacterial DNA contaminants present in commonly used DNA extraction and isolation reagents and demonstrate the significant impact they may have upon investigations of the microbiota and microbiome.

## Methods

### Patient populations and samples

Institutional Review Board approval was obtained at all cooperating Institutions prior to study initiation. Patients with Crohn’s disease and non-inflammatory bowel disease (non-IBD) controls scheduled for surgical resections were recruited from the University of Louisville, Kentucky, and Texas Tech University Health Sciences Center, El Paso, and affiliated Hospitals. A 1 cm^2^ full-thickness section of diseased and/or normal intestinal tissue was obtained under sterile conditions at the time of surgery. Peripheral blood was collected under aseptic conditions in PreAnalytiX PAXgene Blood DNA Tubes (Qiagen, USA) from each patient either at the time of informed consent, during surgical prep, or during surgery. Blood and intestinal tissues were obtained from selected patients previously reported [8].

### DNA isolation kits

The MoBio PowerMax® Soil DNA Isolation Kit 12,988-10 (MoBio Laboratories, USA) was used for all tissue DNA isolations. The moBio Soil DNA isolation kit was chosen because it was the extraction method used by the Human Microbiome Project.

Because our laboratory uses many of these kits on a continuous basis, multiple kits are ordered simultaneously (as many as 10 at a time) and often represent several lot numbers. Since each component within each kit lot has an individual lot number and the individual lot numbers often vary within kits of the same lot, and because several entire kits were often used in a single day and henceforth mixed, no efforts were made to keep track of all the various individual lot numbers. DNA was extracted from blood collected in PreAnalytiX PAXgene Blood DNA Tubes with the PreAnalytiX PAXgene™ Blood DNA Kit (Qiagen, USA). As with the MoBio Kits, the Blood DNA Kits contained kit lot numbers as well as different lot numbers for all the individual kit components.

### Tissue processing and DNA extraction

Methods employed for the processing of tissues, separation of mucosal and submucosal tissues, and extraction of DNA have previously been described [8]. Briefly, after intestinal mucosal and submucosal layers were excised and separated, DNA was extracted from mucosal digests and submucosal tissue using the MoBio PowerMax Soil DNA Isolation Kit employing 100 µm molecular biology grade (MBG) Zirconium beads rather than the supplied garnet beads, followed by digestion with proteinase K, and the use of a high-energy cell disrupter as previously described [8].

DNA was extracted from blood using the PreAnalytiX PAXgene™ Blood DNA Kit following the manufacturer’s protocol. Indigenous DNA contamination was monitored by replacing tissue with 1 ml of MBG water and 100 µl 1 M DL-dithiothreitol, and blood with 5 ml of MBG water and processed as above for the respective tissue type. All tissue and DNA processing were performed under aseptic conditions.

The total amount and purity of DNA present following extraction was determined by spectrometry at 260 and 230 nm in a NanoDrop spectrophotometer. The amount of human DNA in samples was determined using the Quantifiler^®^ Human DNA Quantification Kit (Applied Biosystems) based on the human telomerase reverse transcriptase (hTERT) gene per manufacturer’s instructions.

## 16S Microbiota sequencing on the Illumina MiSeq platform

Bacterial species and microbial ecology within tissue samples and reagents were detected and identified using the 16S universal Eubacterial primers 27Fmod and 519Rmod in the Illumina MiSeq platform with methods based upon the bTEFAP^®^ process [8]. The Q25 sequence data derived from the sequencing were processed using a standardized analysis pipeline [8]. Operational taxonomic units (OTU’s) were defined after removal of singleton sequences, clustering at 3 % divergence (97 % similarity). Final OTUs were taxonomically classified using BLASTn against a curated database derived from GreenGenes Version 13.5 (http://www.greengenes.lbl.gov/cgi-bin/nph-index.cgi), RDPII (http://www.rdp.cme.msu.edu), and NCBI (http://www.ncbi.nlm.nih.gov) databases (including non-bacterial sequences) and compiled into each taxonomic level by both “counts” (actual number of sequences) and “percentage” (relative proportion of sequences within each sample) files.

OTU’s were assigned taxonomic classification based on standard algorithms using the following taxonomic designation: >97 % identity was classified at the species level; between 97 and 95 % identity was designated as an unclassified species; between 95 and 90 % identity was designated as an unclassified genus; between 90 and 85 % identity was designated as an unclassified family; between 85 and 80 % identity was designated as an unclassified order; between 80 and 77 % identity was designated as an unclassified phylum. OTU’s that failed to match any bacterial sequence at 77 % or above, were then blasted against non-bacterial databases to produce alignments and identities within the Metazoa, Bacteria, Fungi, and Viridiplantae Kingdoms as well as sequences classified as Unclassified (less than 77 % match with any database sequence).

### *q*PCR

We attempted to quantify the total bacterial load within samples (both tissue and reagent) using several rRNA gene universal primer sets and probes previously described [7, 10, 15, 16]. Real-time quantitative PCR was performed using the Applied BioSystems Taqman Universal Master Mix in an Applied BioSystems Viia-7 Real-Time PCR System using 20 µl total reaction mixture in 384-well plates according to the manufacturer’s instructions. PCR conditions were 50 °C for 2 min, 95 °C for 10 min, and then 40 cycles at 95 °C for 15 s and 60 °C for 1 min. The Qiagen QuantiFast Pathogen PCR + IC Kit was also evaluated according to the manufacturer’s instructions. All plates were inoculated with an EpMotions 7075 robotic liquid handling system in 384 well plates. Tissue DNA was normalized to 50 ng/µl, based on optical density in a Nanodrop spectrophotometer, previously determined to be an appropriate concentration in most *q*PCR applications [17].

Data were analyzed using the Applied BioSystems Viia-7 software. All assays were performed in triplicate and included negative controls without patient template DNA (no template controls, NTC). A strain of enteroaggregative (EAEC) *Escherichia coli* (strain 042) was used as a positive control and as a quantitative standard in all assays. No template controls with only MBG water and no template were used to detect bacterial DNA in the *q*PCR protocol and reagents.

To eliminate the MGB water as the source of bacterial contamination, MGB water used as a negative control in all assays was exposed to UV radiation for at least 12 h (overnight) prior to use.

### Removal of contaminating DNA

Since treatment with DNAse, restriction endonuclease digestion, and UV radiation have in the past proved unsuccessful in eliminating background DNA from PCR reagents without compromising the subsequent PCR reaction [7], we attempted to use ethidium monoazide (EMA) treatment of our PCR master mixes, water, and reagents as previously described [10, 11, 18]. This method had previously been purported to be effective in determining bacterial loads in plasma.

Briefly, master mixes, MGB water, and other reagents used in the *q*PCR reaction were treated with various concentrations of EMA and exposed to a 500 W halogen light at 20 cm distance on ice for various time periods to determine the optimal EMA concentrations and light exposure times. Optimal EMA concentrations and light exposure times were determined for each reagent lot.

### Statistical analysis

Statistical analysis was performed using a variety of computer packages including XLstat, NCSS 2007, “R” (http://www.r-project.org/) and NCSS 2010 as previously described [8]. Significance reported for any analysis was defined as p < 0.05, corrected for multiple testing using ANOVA with Tukey’s HSD (honestly significant difference) post hoc analysis.

## Results

### Bacterial quantitation based on 16S rRNA sequencing

We first sought to estimate the amount of human and bacterial DNA in each of the samples by comparing the alignment of OTU’s to the Bacteria and Metazoan kingdoms. Since it requires 1000 bacterial genomes to equal a single human genome [19], a sample containing an abundance of human DNA would be considered to have a low microbial biomass. Although only semi-quantitative [12], the number of read counts (fasta hits) and the alignment of OTU’s can be indicative of the overall relative abundance of bacteria DNA as well as the amount of competing human DNA.

Illumina MiSeq 16S rRNA gene sequencing of blood generally produced an average of only ~2000 sequences per sample (as opposed to concurrently processed mucosal tissues which yielded on average ~116,000 sequences). Furthermore, only about 25 % of the generated OTU’s in blood aligned to the Kingdom *Bacteria*, with the remainder 75 % aligning with Metazoa, suggested a low microbial biomass and high metazoan (human) DNA. Similar observations were noted with submucosal tissues. Where mucosal tissues averaged 116,248 sequences per sample, the subjacent submucosal samples (run concurrently) averaged only 15,461 sequences. There was also a significant difference in the number of sequences aligning to the human genome (p ≤ 0.001) between the mucosa and submucosa confirming that submucosal samples, similarly to blood, had a high human DNA relative content and a low microbial biomass.

### *q*PCR using universal rRNA gene primers and probes

In an effort to determine the total bacterial load present within peripheral blood and submucosal tissues of patients with Crohn’s disease, we evaluated the use of *q*PCR using universal rRNA gene primers and Taqman probes. Quantitation, however, generally proved unsuccessful in DNA from blood, submucosal tissues, and other low bacterial biomass samples because C_T_ values were often equal to or greater than those produced in no template controls. No template controls, using irradiated MBG water instead of template, typically produced C_T_ values averaging 29 (range 26–31).

In an effort to reduce the background effects contamination, we evaluated a variety of different universal primers and *q*PCR master mixes. There was no statistically significant difference in the level of background microbial DNA detected in no template controls (NTC) between the different universal rRNA gene primer-probes evaluated; however, the universal primers and probe with additional forward primers for *Propionibacterium* and reverse primer for *Bacteroides*, as previously described [10], consistently produced lower C_T_ values and were used in most subsequent experiments. There was also no significant difference in the level of amplification (C_T_ values) in no-template controls using master mixes from different manufacturers, even those claimed to have low DNA contamination and designed specifically for bacterial DNA quantitation.

**Fig. 1:**
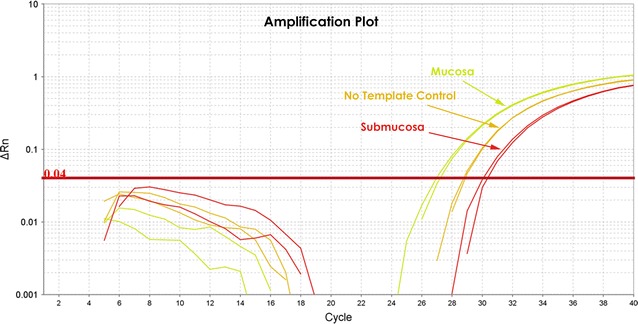
Contaminating endogenous DNA from DNA extraction and processing kits produced C_T_ values of 28–30 in no template controls using universal rRNA gene primers. Samples containing low bacterial biomass and high levels of competing human DNA, such as intestinal submucosal or peripheral blood samples, often produce C_T_ values greater than the no template controls. Bacteria are discernable from background only in samples containing a high bacterial biomass such as intestinal mucosal tissues. Reagent contamination interferes and prevents bacterial quantitation based on the rRNA genes in low bacterial biomass samples

Samples containing large amounts of human DNA and small amounts of microbial DNA as estimated by fasta counts and OTU alignments, such as submucosal tissues and blood, produced C_T_ values greater than no template controls suggesting that there was less bacteria in the tissue samples than in negative controls (Fig. 1). As such, this background precluded bacterial quantitation with *q*PCR using universal primers. This inhibition (C_T_ value greater than no template controls) was presumed to be the result of competitive inhibition created by the large amount of human DNA in the sample [20]. Bacterial quantitation could only be achieved in mucosal samples which consistently produced C_T_ values less than negative template controls allowing the amount of bacterial DNA present in the sample to be estimated based on internal bacterial standards.

### Removal of inherent microbial DNA contamination from PCR reagents

Since background contamination precluded bacterial quantitation, we sought to remove the background bacterial DNA in anticipation that such would allow quantitation of submucosal and blood bacterial DNA levels. Pre-treatment of master mixes, primers and probes, and MBG water (for NTC controls) with pre-optimized concentrations of EMA, eliminated or greatly reduced background contaminating microbial DNA but caused a 3–4 cycle downward shift in C_T_ values which adversely affected detection, quantitation, as well as reproducibility and uniformity (standard deviation) of samples. Thus, pre-treatment of reagents with EMA was found to be ineffective in blood and submucosal samples making efforts to eliminate background DNA technically difficult and unproductive.

### Quantitation of contaminating bacterial DNA

Since *q*PCR suggested that tissue and blood samples contained less bacterial DNA than negative controls (CT values greater than no template controls), efforts were made to quantify the amount of bacterial DNA in negative controls. Such determinations could allow the quantitation of bacterial DNA in tissue samples by using negative controls as standards in quantitation by ΔΔCT.

Spectrophotometric analysis of the PreAnalytiX PAXgene™ Blood DNA Kit using MBG water instead of blood failed to yield any detectable DNA or the amount of DNA detected was negligible (<1 ng/µl). The processing of MBG water through the MoBio PowerSoil DNA Isolation Kit typically yielded an optical density (OD) reading at 260 nm suggesting 19–25 ng DNA per µl. The 260/280 OD ratio was, however, always >2.0 and produced a flattened spectral peak suggesting the presence of contaminants affecting the absorbance at 260 nm. These findings suggest that OD readings obtained following extraction with the MoBio PowerSoil DNA kit may not be accurate and may over estimate the amount of DNA in low biomass samples.

**Fig. 2:**
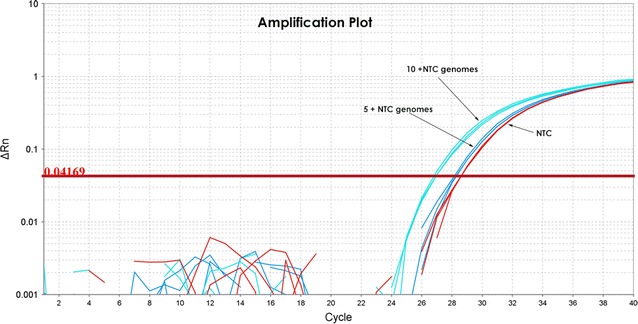
Background levels of bacterial genomes based on 16S rRNA gene universal primers suggest a level of at least 4 bacterial genomes per µl of *q*PCR reaction mixture. Although the true amount of contaminating bacterial DNA would be 4 + NTC, assuming the lowest possible figure, a standard 50 µl *q*PCR reaction would contain at least 200 *E. coli*-equivalent genomes or approximately 1400 rRNA gene copies contaminating the reaction

To more accurately determine the amount of DNA present after processing MBG water through the DNA extraction kits, we performed quantitative *q*PCR using serial dilutions of pure microbial DNA derived from *E. coli*. Assuming the complete absence of contamination, the copy number of 16S rRNA genes should correlate with the dilutions of *E. coli* and reduce or increase in a linear fashion as a standard curve. C_T_ values remained stable and did not reduce further at dilutions below 5 bacterial genomes per µl, indicating the presence of background DNA at approximately 28–35 rRNA copies per µl (4–5 *E. coli* genomes) in both the Applied Biosystems and the Qiagen Master Mixes (Fig. 2). This contamination estimate was based on the assumption that there was a complete absence of contamination and that the C_T_ values of standards were not influenced or altered by background contaminating DNA levels. Thus, the true level of DNA contamination would be 28–35 rRNA copies per µl + unknown contaminating genomes in the *q*PCR master mix.

Having determined the amount of contamination present in negative PCR controls, we could now determine the amount of contamination that came from DNA extraction kits as opposed to other sources such as PCR reagents, laboratory consumables, or lab personnel. Processing purified MBG water through the MoBio PowerSoil DNA extraction kit followed by *q*PCR with universal bacterial primers, based on calculation of bacterial DNA on quantitative *E. coli* standards and relative ΔΔC_T_, it was estimated that the MoBio PowerSoil DNA extraction kit contributed approximately 10–15 *E. coli* equivalent genomes (70–105 rRNA gene copies) per µl of template to the *q*PCR reaction mixture.

### Influence of competitive DNA on background DNA levels

The amount of contaminating DNA calculated above (Fig. 2) was based on the addition of pure *E. coli* bacterial DNA in MBG-irradiated water and does not consider the effects of human or other DNA as a competitive inhibitor of the PCR reaction, as would be present in most clinical samples [20]. To determine the effect of human DNA on sensitivity (competitive inhibition) and obtain more accurate bacterial quantitation, we added 250 ng of human peripheral blood DNA (50 ng/µl in 5 µl) and increasing amounts of *E. coli* genomic DNA to ascertain the amount of bacterial DNA required to exceed background in the presence of an excess amount of human DNA. As done previously, assuming a complete absence of contamination, the C_T_ value corresponding to the number of 16S rRNA gene copies present in the sample should correlate with dilutions of *E. coli* and reduce in a linear manner as a standard curve.

At 125 *E. coli* genomes, equivalent to 875 rRNA gene copies, the copy number (C_T_ value) remained stable and did not reduce further, indicating that approximately 875 rRNA gene copies (as opposed to 35 rRNA gene copies in water) were required to overcome the inhibitory effects of peripheral blood DNA. These findings support the notion that the true amount of contaminating bacterial DNA is far greater than the 4–5 genomes calculated based on pure bacterial DNA above. Because the peripheral blood DNA did not display any inhibitory effects with primers/probes directed against the human genome, including the human telomerase reverse transcriptase used in DNA quantitation, the inhibition observed is presumed to result from competitive inhibition created by the excess amount of human DNA in the samples.

### Diversity of contaminating bacterial DNA

Illumina MiSeq 16S rRNA gene microbiota sequencing identified a host of contaminating bacterial taxa within *q*PCR no template controls and DNA extraction kits. We identified 88 additional genera that may be found as contaminants of commonly used DNA extraction kits, bringing the total of known contaminating genera to 181 (Tables 1, 2; Additional file 1: Table S1). By sequencing negative PCR controls and irradiated-MBG water we are able to differentiate what came from DNA extraction kits as opposed to other sources including reagents, laboratory consumables, or laboratory personnel.

**Table 1:** Bacterial families and genera identified in peripheral blood DNA extraction kits processed with molecular biology grade water instead of blood

Family	Genus	Relative %^a^
*Actinomycetaceae*	*Actinomyces* spp.	0.082576
*Bacillaceae*	*Geobacillus* spp.	56.39967
*Bacteroidaceae*	*Bacteroides* spp.	0.743187
*Bifidobacteriaceae*	*Bifidobacterium* spp.^b^	0.082576
*Carnobacteriaceae*	*Granulicatella* spp.^b^	0.165153
*Comamonadaceae*	*Alicycliphilus* spp.^b^	0.082576
	*Pelomonas* spp.	3.220479
*Coriobacteriaceae*	*Atopobium* spp.^b^	0.082576
*Enterobacteriaceae*	*Escherichia* spp.	0.247729
*Enterococcaceae*	*Enterococcus* spp.^b^	0.165153
*Erysipelotrichaceae*	*Erysipelatoclostridium* spp.^b^	0.082576
*[Gemellaceae]*	*Gemella* spp.^b^	0.082576
*Lachnospiraceae*	*Blautia* spp.	1.156069
	*Dorea* spp.	0.247729
	*Roseburia* spp.	0.247729
*Lactobacillaceae*	*Lactobacillus* spp.^b^	0.082576
*Microbacteriaceae*	*Agrococcus* spp.	0.247729
*Oxalobacteraceae*	*Herbaspirillum* spp.^b^	0.082576
*Pasteurellaceae*	*Haemophilus* spp.	0.495458
*Phyllobacteriaceae*	*Phyllobacterium* spp.^b^	0.082576
*Porphyromonadaceae*	*Parabacteroides* spp.^b^	0.082576
*Propionibacteriaceae*	*Propionibacterium* spp.	34.10405
*Pseudomonadaceae*	*Pseudomonas* spp.^b^	0.082576
*Rhodobacteraceae*	*Ruegeria* spp.^b^	0.082576
*Rikenellaceae*	*Alistipes* spp.^b^	0.082576
*Streptococcaceae*	*Streptococcus* spp.	1.156069
*Veillonellaceae*	*Dialister* spp.	0.082576
	*Quinella* spp.	0.082576
	*Veillonella* spp.	0.165153

Bacterial DNA belonging to 29 genera and 39 tentative species were found to contaminate peripheral blood DNA extraction kits

See Additional file 1: Table S1 for list of tentative species identification

^a^Relative percent prevalence are average of multiple lots

^b^Detection of organism was lot dependent and not present in all lots

**Table 2:** Bacterial families and genera identified within the MoBio DNA extraction kit DNA from 81 bacterial genera and 108 tentative species were identified as inherent contaminants

Family	Genera	Relative %^a^
*Acetobacteraceae*	*Roseomonas*	1.71
*Actinomycetaceae*	*Actinomyces*	0.03
*Aerococcaceae*	*Abiotrophia*	1.13
*Alcanivoracaceae*	*Alcanivorax* ^b^	0.005
*Alicyclobacillaceae*	*Tumebacillus*	3.7
*Alphaproteobacteria, unclassified*	*Candidatus_alysiosphaera* ^b^	0.003
*Aurantimonadaceae*	*Aurantimonas* ^b^	0.002
*Bacillaceae*	*Bacillus*	0.3
*Bacillales family xi. incertae sedis*	*Gemella* ^b^	0.005
*Bacteroidaceae*	*Bacteroides*	0.08
*Bifidobacteriaceae*	*Bifidobacterium* ^b^	0.003
*Bradyrhizobiaceae*	*Afipia* ^b^	0.001
	*Bradyrhizobium*	0.51
*Burkholderiaceae*	*Burkholderia*	0.12
	*Roseateles*	1.31
*Cardiobacteriaceae*	*Cardiobacterium*	0.014
*Carnobacteriaceae*	*Granulicatella*	0.02
	*Trichococcus* ^b^	0.002
*Christensenellaceae*	*Christensenella* ^b^	0.002
*Clostridiaceae*	*Clostridium*	0.024
*Clostridiales, unclassified*	*Flavonifractor* ^b^	0.002
	*Pseudoflavonifractor* ^b^	0.002
*Clostridiales family xi. incertae sedis*	*Anaerococcus* ^b^	0.004
*Comamonadaceae*	*Comamonas*	0.037
	*Coprococcus* ^b^	0.002
	*Curvibacter*	0.155
	*Pseudorhodoferax*	0.32
*Coriobacteriaceae*	*Atopobium* ^b^	0.002
	*Collinsella* ^b^	0.005
*Corynebacteriaceae*	*Corynebacterium*	2.2
*Eggerthellaceae*	*Eggerthella* ^b^	0.007
	*Slackia* ^b^	0.002
*Enterobacteriaceae*	*Escherichia*	0.03
	*Escherichia_Shigella* ^b^	0.003
	*Klebsiella*	0.02
	*Serratia*	0.007
*Enterococcaceae*	*Enterococcus*	0.008
*Erysipelotrichaceae*	*Catenibacterium* ^b^	0.002
	*Erysipelatoclostridium*	0.02
	*Solobacterium* ^b^	0.005
	*Turicibacter*	0.021
*Eubacteriaceae*	*Eubacterium* ^b^	0.003
*Flavobacteriaceae*	*Capnocytophaga*	0.21
	*Chryseobacterium*	0.33
	*Cloacibacterium*	0.003
*Fusobacteriaceae*	*Fusobacterium*	0.28
*Geodermatophilaceae*	*Blastococcus*	0.25
*Gordoniaceae*	*Gordonia*	0.02
*Halanaerobiaceae*	*Halocella* ^b^	0.005
*Lachnospiraceae*	*Blautia*	0.02
	*Johnsonella* ^b^	0.002
	*Lachnoanaerobaculum*	0.23
	*Lachnoclostridium* ^b^	0.005
	*Lachnospira*	0.007
	*Moryella* ^b^	0.002
	*Pseudobutyrivibrio*	0.02
	*Roseburia*	0.009
	*Tyzzerella* ^b^	0.002
*Lactobacillaceae*	*Lactobacillus*	0.009
*Leptotrichiaceae*	*Leptotrichia*	0.28
*Methylobacteriaceae*	*Methylobacterium*	11.7
*Methylocystaceae*	*Methylopila* ^b^	0.003
*Microbacteriaceae*	*Pseudoclavibacter* ^b^	0.005
*Micrococcaceae*	*Arthrobacter*	0.03
	*Rothia* ^b^	0.002
*Micromonosporaceae*	*Pilimelia* ^b^	0.002
*Moraxellaceae*	*Enhydrobacter*	1.95
*Neisseriaceae*	*Kingella* ^b^	0.002
	*Neisseria*	0.05
*Oxalobacteraceae*	*Janthinobacterium*	0.002
	*Massilia*	0.11
	*Oxalobacter*	2.3
*Paenibacillaceae*	*Brevibacillus*	0.03
	*Paenibacillus* ^b^	0.003
*Pasteurellaceae*	*Haemophilus*	0.26
*Pelagibacteraceae*	*Candidatus pelagibacter* ^b^	0.002
*Peptococcaceae*	*Peptococcus* ^b^	0.003
*Peptoniphilaceae*	*Parvimonas*	0.02
	*Peptoniphilus* ^b^	0.002
*Peptostreptococcaceae*	*Intestinibacter*	0.043
*Phyllobacteriaceae*	*Phyllobacterium*	0.3
*Porphyromonadaceae*	*Porphyromonas* ^b^	0.003
*Prevotellaceae*	*Prevotella*	10.4
	*Xylanibacter* ^b^	0.002
*Propionibacteriaceae*	*Propionibacterium*	15.8
*Pseudomonadaceae*	*Pseudomonas*	0.009
*Rhodobacteraceae*	*Rubellimicrobium*	0.78
*Rikenellaceae*	*Alistipes*	0.05
*Ruminococcaceae*	*Anaerotruncus* ^b^	0.001
	*Faecalibacterium*	0.02
	*Fastidiosipila* ^b^	0.003
	*Oscillospira* ^b^	0.002
	*Papillibacter* ^b^	0.003
	*Ruminiclostridium* ^b^	0.003
	*Ruminococcus*	0.007
*Sphingobacteriaceae*	*Sphingobacterium* ^b^	0.003
*Staphylococcaceae*	*Staphylococcus*	5.2
*Streptococcaceae*	*Streptococcus*	13.4
*Sutterellaceae*	*Parasutterella* ^b^	0.005
*Thermaceae*	*Meiothermus*	0.06
*tm7 (candidate division)*	*tm7 (candidate division)*	0.44
*Veillonellaceae*	*Dialister* ^b^	0.003
	*Megasphaera*	1.53
	*Selenomonas*	0.44
	*Veillonella*	20.4
*Xanthomonadaceae*	*Stenotrophomonas*	1.25

These taxa are unlikely to have been introduced by PCR kit reagents or contamination on the bench, and so are likely to have originated from the DNA extraction kit and during the DNA extraction process

See Additional file 1: Table S1 for list of tentative species identification

^a^Relative percent prevalence are average of multiple lots

^b^Detection of organism was lot dependent and not present in all lots

#### Blood

Sequencing of MBG water that was processed through the Qiagen PreAnalytiX PAXgene™ Blood DNA Kit and sequencing the end product on the Illumina MiSeq platform generated an average of 1209 sequences which translated into 44 operational taxonomic units (OTU’s) belonging to the Kingdom *Bacteria* and aligning to 24 bacterial families, 29 genera and 39 tentative species (Table 1). Of the 29 Genera, 13 were present at a relative prevalence >0.1 %. Among the tentative species identified, *Geobacillus thermoparaffinivorans* accounted for 54 % of the relative population, followed by *Propionibacterium acnes* at 34 %, and *Pelomonas* spp. at 3 %. The remaining 35 species were present at a relative prevalence of <1 %, but included many species commonly associated with the environment and the gastrointestinal microbiota (Additional file 1: Table S1).

#### Tissue

Sequencing of MBG water processed through the MoBio Soil DNA Isolation Kit and sequencing the end product on the Illumina MiSeq platform produced an average of 29,387 sequences, suggesting an abundance of bacterial DNA. These sequences formed 1618 OTU’s of which 54 OTU’s did not align with any known sequence (no hits found) within the Kingdoms Metazoa, Bacteria, Fungi or Viridiplantae. In total, DNA from 81 bacterial genera and 108 tentative species were identified as contaminants within this isolation kit (Table 2). Organisms of the genera *Propionibacterium* spp. (26 %) and *Methylobacterium* spp. (22 %) accounted for almost 50 % of the population, although a variety of other organisms were represented at relative frequencies >1 %. Of the 108 tentative species of bacterial DNA detected, 74 % or 80 species are specifically associated with and considered normal inhabitants of the human gastrointestinal tract and/or the environment (Additional file 1: Table S1).

### Distortion of taxonomic distributions and relative frequencies by contaminating bacterial DNA in low microbial biomass samples

#### Peripheral blood

Although the total number of sequences and OTU’s generated from blood samples were greater than blood blank controls (MBG water processed through the DNA extraction/isolation methods), the actual sequence counts (~1000) and OTU’s that corresponded to bacteria were generally equal to or less than blank controls, presumably the result of competitive inhibition by large excesses of human DNA. The OTU’s that misaligned with human DNA represented on average 76 % of all generated OTU’s. Although 176 discrete bacterial genera were identified within blood samples from patients with Crohn’s disease and controls in total, many genera were unique to one or more individuals.

The predominant genera found in blood *blank controls* were *Geobacillus* (~56 %), *Propionibacterium* (~34 %), and *Pelomonas* (~3 %) with remaining bacteria representing <1 % relative prevalence (Table 1). Although rRNA genes of *Propionibacterium* spp*. and Pelomonas* spp. were found in peripheral blood at low relative prevalence (~1 and ~0.05 %, respectively), organisms of the genus *Geobacillus* were completely absent in all intestinal tissue samples previously examined [8], representing 70 DNA samples, and only detected in 3 blood samples. Based on the presence of *Geobacillus* spp. within blood samples, the relative prevalence of bacteria in these samples were considered to be possibly distorted by contaminating DNA from the extraction kit.

Removing bacteria of low prevalence (<0.01 % relative prevalence) and in less than 50 % of the patient population (~92 % relative prevalence remaining) and then removing bacteria identified in blank controls as possible contamination left only 5 species remaining: *Anaerostipes* spp., *Mogibacterium* spp., *Subdoligranulum* spp., *Halocella* spp., and *Sphingobium* spp. that collectively represented less than 1 % relative prevalence in the original blood sample. Thus, elimination of bacteria that were identified as possible contamination in blank controls was not a viable solution of dealing with possible contamination issues.

#### Tissue samples

DNA isolated from submucosal tissues contained significantly more sequences that aligned with human DNA (similar to blood) than the corresponding DNA isolated from the superjacent mucosa (p ≤ 0.001) and more closely resembled peripheral blood than intestinal mucosal tissues. The generated metazoa:bacteria ratio of the submucosal sequences averaged 2.7, similar to blood at 3.6 and dissimilar to most mucosal samples which averaged 0.003. These findings support the notion that DNA extracted from submucosal tissues contains a high human DNA content and a low microbial biomass.

The total number of sequences generated, i.e., depth of coverage, was not a reliable indicator of bacterial content. Although the total number of sequences generated averaged 67,500 per intestinal submucosal sample, the total number of sequences that aligned with the Kingdom *Bacteria* was 22,800 (as opposed to 102,655 for the superjacent mucosa). This was often less than that of tissue extraction blank controls (which averaged 23,845) run concurrently.

Determining the effects of microbial contamination of reagents on submucosal tissue samples proved more problematic than with peripheral blood samples. The predominate organisms present in tissue extraction *blank controls* were organisms of the Genera *Corynebacterium* (~2 %), *Methylobacterium* (~12 %), *Prevotella* (~10 %), *Propionibacterium* (~16 %), *Staphylococcus* (~5 %), *Streptococcus* (~13 %), and *Tumebacillus* (~4 %) which represented approximately 70 % relative frequency of contaminating DNA from the extraction kits. These and most other bacteria present within extraction controls are considered normal and common inhabitants of the intestinal tract and were detected in most submucosal and mucosal tissue samples. As with peripheral blood, removal of contaminating genera from submucosal tissue samples resulted in only 5 % of the relative bacterial population remaining. Most other organisms within tissue extraction blanks were present in low relative prevalence (<0.1 %) and lot dependent. Evaluating the effects of contamination in tissue samples required analysis at the tentative species level.

Identification at the species level based on rRNA gene sequences can only be considered tentative due to overlapping rRNA genes between species and the ever-changing bacterial classification at the species level. Nevertheless, run concurrently, differences at the species level can be used to determine whether inherent contamination was likely to have influenced microbial relative prevalence within low bacterial biomass samples. However, each reagent(s) lot needed to be sequenced in addition to being sequenced concurrently with each sample run to compensate for differences in sequencing depth/coverage.

The stone-dwelling *Actinobacteria*, *Blastococcus saxobsidens*, was found to be a consistent organism detected as contaminating DNA from all tissue extraction *control blanks* and, being aerobic and a having a preferred growing temperature of 28 °C, is not generally considered a normal inhabitant of the gastrointestinal tract. It was not detected in 145 mucosal samples or 47 blood samples. It was detected in only 6 of 142 submucosal samples. All six of the submucosal samples in which *B. saxobsidens* was detected also contained DNA from other unexpected bacteria found in blank controls including one or more of *Arthrobacter psychrolactophilus, Meiothermu*s spp., *Methylobacterium radiotolerans, Roseateles depolymerans*, and *Roseomonas cervicalis*. Based on the presence of DNA from these species within submucosal tissues, the bacterial relative prevalence in these samples were considered to be possibly distorted by contaminating DNA from the extraction kits.

### Similarities between DNA extraction Kits

Although there was diversity in the number of genera (19–24) and species (78–108) between lots of the MoBio DNA Extraction Kit, the predominant 20 bacterial genera (and 20 bacterial species) representing 69 % (range 65–72 %) of the relative bacterial population were consistently detected in all lots. Diversity in the other 31 % (represented in 88 genera and 143 species) were present in low prevalence and were lot dependent. Contaminants in the Qiagen Blood Kit, although comprising on average 29 distinct genera and 39 species, was predominately composed of *Propionibacterium acnes* and *Geobacillus thermoparaffinivorans* which collectively represented 91 % of the total relative prevalence, 34 and 57 % respectively.

There were limited similarities in the bacterial contaminants detected between the MoBio and Qiagen Kits. Although ~16 % (18 genera and species) were randomly shared between the 2 kits, these were all lot dependent and, except for *Propionibacterium acnes* which was detected in all kits, none of the predominant bacteria were shared between the 2 kits.

## Discussion

It has long been documented that reagents, even molecular biology grade used in the isolation and processing of DNA, can be contaminated with bacterial DNA. Despite this purported general knowledge and high profile reports of reagent contamination affecting microbial datasets and their interpretation [21–23], few studies seem to appreciate the impact such contamination may have on microbiota and other microbial analyses and subsequent conclusions. Although contamination from exogenous or inherent sources may not be a problem in samples containing a high microbial load, many environmental and human tissue samples have low bacterial biomass in relation to the overall sample size and DNA content.

As demonstrated herein, inherent contamination prevented the determination of bacterial load within samples containing low microbial content such as in human intestinal submucosal and peripheral blood samples. Because of background contamination, our threshold of detection (C_T_) using pure bacterial DNA in water was approximately 10 *E. coli* equivalent genomes or approximately 70 rRNA gene copies per µl in the absence of competing human DNA. Detection levels were significantly affected by the presence of competing human DNA, increasing to 125 *E. coli* genomes or 875 rRNA gene copies per µl to overcome background contamination.

Salter et al. [12] reported, using spiked *Salmonella bongori* as template, that at least 1000 *S. bongori* genomes were required to obtain reliable rRNA gene sequences and that at template concentrations less than 1000, sequences were dominated by the contaminating microbial DNA. This is similar to our findings. These investigators also suggested that the FastDNA Spin Kit for Soil (MP Biomedicals, Santa Anna, CA) may contribute as many as 500 rRNA gene copies per µl of elusion buffer [12]. In contrast, although present, we were unable to determine the amount of contaminating DNA in the Qiagen Blood Kit and only 10–15 *E. coli* genome equivalents (70–105 rRNA gene copies) per µl elution buffer from the MoBio Power Soil DNA Extraction Kit. The FastDNA Spin Kit for Soil contained 63 bacterial taxa having a relative prevalence >0.1, while we only detected 41 total bacterial taxa (16–25 taxa per lot) having a relative prevalence >0.1 in the MoBio Kit (78–108 total taxa) and only 17 taxa in the Qiagen Kit (39 total taxa). Thus, the amount of contaminating DNA may vary greatly depending on the manufacturer and the specific DNA extraction kit used.

As shown with the MoBio Power Soil DNA Kit, processing irradiated MBG water through the kit and checking the DNA elusion by spectrophotometry can give the impression that the DNA extraction was successful when in fact the DNA that is present and being measured may all be contaminating DNA from the extraction kit. When dealing with samples containing a low bacterial biomass, a single bacterial genome contaminant could greatly alter the relative bacterial prevalence within the sample. It is worth noting that the MoBio Power Soil DNA Kit was the primary DNA extraction method of the Human Microbiome Project [24].

The issue of endogenous bacterial DNA contamination is not limited to rRNA microbiota sequencing, but applies equally to PCR and other genetic determinations. We show that a commonly used DNA extraction kit and master mix would contribute 200–275 *E. coli* equivalent bacterial genomes (2100–2800 rRNA gene copies) in a typical 50 µl *q*PCR reaction. This level of contamination with endogenous bacterial DNA could greatly influence results, particularly if the target of a PCR reaction was present within the contaminating DNA mixture. As such, it is inappropriate to use MBG water as a negative (NTC) control in PCR reactions as false positive reactions could occur due to microbial contamination from the extraction kit and/or PCR master mixes. DNA extraction blanks using the same extraction kit lots and processing reagents need to be used as NTC controls to monitor for target sequences within DNA contamination. The use of MBG water, the typical control, rather than DNA extraction blanks could account for the irreproducibility of results within and between laboratories [25].

Although workflow for post-sequencing processing of data has been suggested and is generally the recommended method used to deal with inherent contamination in samples with low bacterial content [26], these methods rely on discounting low prevalence organisms. Discounting low prevalence organisms, however, may be erroneous. Microbial community profiling based on 16S rRNA genes is fraught with bias created by DNA extraction methods, competitive DNA (human or otherwise), primers used during PCR amplification, the sequencing platform used, depth of coverage, and bioinformatics [20, 27], to name just a few. Depending on the bias, organisms identified as low prevalence could actually be of high relative frequency or be the most significant microbe in the sample and should not be arbitrarily discarded. It is undisputed that *Mycobacterium leprae* is the causative agent of human leprosy, but organisms of the genus *Mycobacterium* appear within the leprous lesion at a relative frequency of 0.0007 % as determined by 16S rRNA microbiota sequencing of leprosy skin lesions [28]. The relative frequency of *M. leprae* in paucibacillary (tuberculoid) leprosy would be substantially less. Under recommendations to discount low prevalence organisms, mycobacteria in general (including *M. leprae*) would be erroneously discounted as insignificant. Thus, arbitrarily discarding low prevalence microbes as a means of compensating for contamination issues may be seriously flawed and prevent the detection of pertinent microbes within environmental and tissue samples.

We routinely perform blank DNA extractions (using irradiated-MBG water rather than tissue) during each tissue extraction to monitor for both endogenous and exogenous contamination. These extraction blanks and water blanks are sequenced side by side with samples so that contamination can be ruled out as the source of unique or unusual findings. However, as noted in Tables 1, 2 and supplemental data, most of the contaminating bacteria are common environmental organisms and/or associated with the gastrointestinal tract or skin. For environmental or tissue samples the problem of identifying contaminants requires special attention as the contaminants may be taxa that are indistinguishable from those genuinely present in the samples. In the samples examined herein, only *Geobacillus thermoparaffinivorans* in the Qiagen kit and *Blastococcus saxobsidens* in the MoBio kit were the only useful indicators of possible contamination.

## Conclusions

Laboratories working on bacterial populations need to define contaminants present in all extraction kits and reagents used in the processing of DNA and make such determinations with each lot and with each processing of samples. Extraction blanks need to be used as no template and other controls as opposed to unprocessed water. Any unusual and/or unexpected findings need to be viewed as possible contamination as opposed to unique findings. Absent consideration and descriptions of methods used to monitor and deal with contamination issues, data from tissue and other low microbial biomass studies, such as the tissue microbiota [29, 30], must be questioned. As noted herein, DNA extraction kits themselves may generate more than 20,000 sequences on the Illumina MiSeq platform representing more than 81 bacterial genera.

## Additional file

10.1186/s13099-016-0103-7 Taxonomic listing of all bacterial genera and species detected as contaminants of DNA extraction and processing kits in the present study and previously reported.
